# Progressive damage mechanism and multiscale characterization of hard sandstone-coal composite structures under loading

**DOI:** 10.1371/journal.pone.0341958

**Published:** 2026-03-13

**Authors:** Jiaxin Dang, Min Tu, Xiangyang Zhang, Qingwei Bu

**Affiliations:** 1 School of Mining Engineering, Anhui University of Science and Technology, Huainan, China; 2 School of Mining and Coal, Inner Mongolia University of Science and Technology, Baotou, China; Guizhou University, CHINA

## Abstract

Under geological conditions characterized by deep high-stress zones, intense mining disturbances, and hard roof strata, primary fractures on and within coal bodies are highly susceptible to expansion and propagation. This study investigates the failure characteristics of coal bodies under the combined effects of hard roof strata and sandstone fracture water. Taking the 11129 working face at Zhangji Coal Mine as the study site, this research examines the macro- and micro-scale load-bearing capacity and damage characteristics of coal bodies. The results indicate that: (1) During coal seam mining, the thick layer of hard sandstone roof directly overlying the seam exhibits a large span with minimal deformation while bearing the load of the overlying weak rock strata. By employing pre-splitting blasting technology, the initial roof fracture step length was reduced to 45 m. Stress concentration occurred 5–20 m ahead of the coal face, with peak stress reaching 33.8 kPa. Peak strain appeared 11m behind the coal face, registering a value of 594.3. (2) During the moment of stress-bearing or fracture, coal-rock strata undergo energy accumulation and dissipation. Before the advance distance reaches the minimum roof fracture step length, relatively hard rock layers bear their own weight and the load from overlying softer strata. As the span length of the hard rock increases, the accumulated energy rises. When the internal energy accumulation reaches the rock’s load-bearing limit, the roof fractures, transferring the force to the goaf and releasing the energy. (3) Under dynamic loading, coal sample cracks exhibit a progressive evolution from “development to penetration to failure.” At low impact intensity (0.3 MPa), the incident energy for samples with varying coal-to-rock ratios ranges from 172.64 to 240.89 J, with dissipated energy accounting for 0.249 to 0.44 of the total. When the impact pressure increased to 0.7 MPa and the rock content in the sample rose, the incident energy increased from 265.95 J to 326.87 J, an increase of approximately 60.92 J. The proportion of dissipated energy remained within the range of 0.249 to 0.418. The magnitude of the impact pressure had no significant effect on the degree of energy dissipation. (4) A pre-splitting blast pressure relief scheme employing fan-shaped hole groupings was proposed. Numerical results from the working face indicate that the pre-splitting blast achieved the anticipated roof control effect.

## 1. Introduction

The influence of hard roof strata on coal bodies during deep coal mining remains a key research focus. Under deep high-stress conditions and intense mining disturbances, hard roof strata exert pressure on coal bodies. This causes the expansion and elongation of primary fractures both on the coal surface and within the coal body, leading to coal spalling and fracturing. Therefore, understanding the load-bearing characteristics of coal bodies and the fracture development patterns during the rotational subsidence of hard roof strata is crucial for safe and efficient coal seam mining [1–6].

Domestic and international scholars have conducted extensive research on the extent to which hard roof strata affect coal-bearing rock masses during deep coal mining operations [7–10]. Dong X. et al. [11] investigated the failure mechanisms of composite hard roof strata. They proposed a bidirectional high-low blasting technique for rock control based on the fracture mechanism of composite hard roof strata. The effectiveness of this method was validated through field microseismic monitoring data. Ren H. et al. [12] assessed the likelihood of coal bursts occurring in deep coal mining workfaces. A mechanical model was established to investigate the mechanism of coal bursts in workfaces. Stress and energy criteria for the coal body in the workface were proposed. Klishin I. V. et al. [13] presented a boundary value problem for rock mass stress-strain modeling. They examined different borehole pattern variants for directional hydraulic fracturing in hard roof formations. Chen C. et al. [14] investigated the behavior of overlying strata and stress distribution characteristics under conditions of thick, hard roof plates and thick coal seams. They concluded that stress variations in the upper section were not pronounced, whereas the lower section exhibited significant differences, with numerous intermittent peak points appearing in the stress variation rate curve. Shao L. et al. [15] analyzed the disaster mechanism of gas explosions triggered by hard roof strata. They proposed control methods for hydraulic fracturing of hard roof strata during comprehensive mechanized mining in thick coal seams. Xia B. et al. [16] established a numerical model based on the Material Particle Method (MPM) to reveal the mechanism of ground fracturing. By comparing results with similar physical simulation tests conducted in the same working face, the reliability of the proposed model was validated. Findings indicate that under excavation disturbance, hydraulic fractures are activated and connect with mining-induced fractures, thereby mitigating the impact of concentrated advance stresses on the working face during hard roof collapse.

The above scholars have produced substantial research findings on crack propagation, dynamic stress-strain behavior, and energy dissipation in coal-rock specimens, addressing the hazards posed by hard roof strata [17–20]. However, the extent of crack propagation on coal rock surfaces and the evolution of nearby strain during the period from crack initiation to penetration have not been sufficiently considered. The presence of water in coal samples, as well as crack propagation and strain evolution under varying impact intensities, also exhibit distinct characteristics. Coal mine working faces frequently encounter impacts from geological structures, rock stress, and mining activities. During coal seam extraction, low-lying rock strata suspended over large spans detach from their bedrock, subsequently fracturing and collapsing to impact the coal body. This causes the coal to undergo shock and vibration. As mining progresses along the coal seam’s strike, medium-to-high-lying coal and rock strata successively fracture and destabilize. This instability propagates to the upper fractured block, forming an unstable block hinge structure that further triggers coal body failure. Therefore, understanding the failure characteristics of coal bodies under dynamic loading impacts is crucial for the safe and efficient operation of mining engineering. This paper addresses the dual influence of the directly overlying hard roof and fractured sandstone water in the roof on coal strength at the 11129 working face of Zhangji Coal Mine. It investigates load transfer and energy evolution under the conditions of a directly overlying hard roof. The analysis focuses on the progressive instability characteristics and evolution process of coal and rock in the mining area. Furthermore, from the perspective of energy accumulation, it examines the degree of elastic energy accumulation in strata at different levels and the mechanical causes of mine pressure manifestation. Targeted management technical measures are proposed for addressing severe mine pressure manifestations, providing valuable reference for understanding the fragmentation characteristics of coal under the combined effects of roof rotation and subsidence and sandstone fissure water in the 11129 working face of Zhangji Coal Mine.

## 2. Project overview analysis

The 11129 working face at Zhangjia Coal Mine is the first mining face for the No. 9 coal seam in East Zone 1. The designed recoverable strike length is 1,200 m, with a dip length of 240 m. The working face elevation ranges from −660.1 m to −709.1 m. The No. 9 coal seam comprises two layers: 9−1 and 9−2. The 9−1 coal is semi-dark to semi-bright in appearance, locally containing thin interbedded gangue, with an average thickness of 1.9 m. The 9−2 seam averages 0.6 m thick, with inter-seam distances ranging from 0.5 to 4.4 m (average 2.0 m). The coal seams dip at angles between 2° and 8°, averaging 4°. The immediate roof above Coal Seam 9 consists of 7.1 m of grayish-white quartz sandstone, predominantly medium-grained in texture. The basic roof comprises 8.0 m of siltstone, exhibiting well-developed vertical joints throughout. Overlying lithologies are primarily sandy mudstone. The immediate footwall of Coal Seam 9 is 2.5 m of mudstone. The basic footwall consists of 5.2 m of siltstone interbedded with two layers of carbonaceous mudstone.

**Fig 1:**
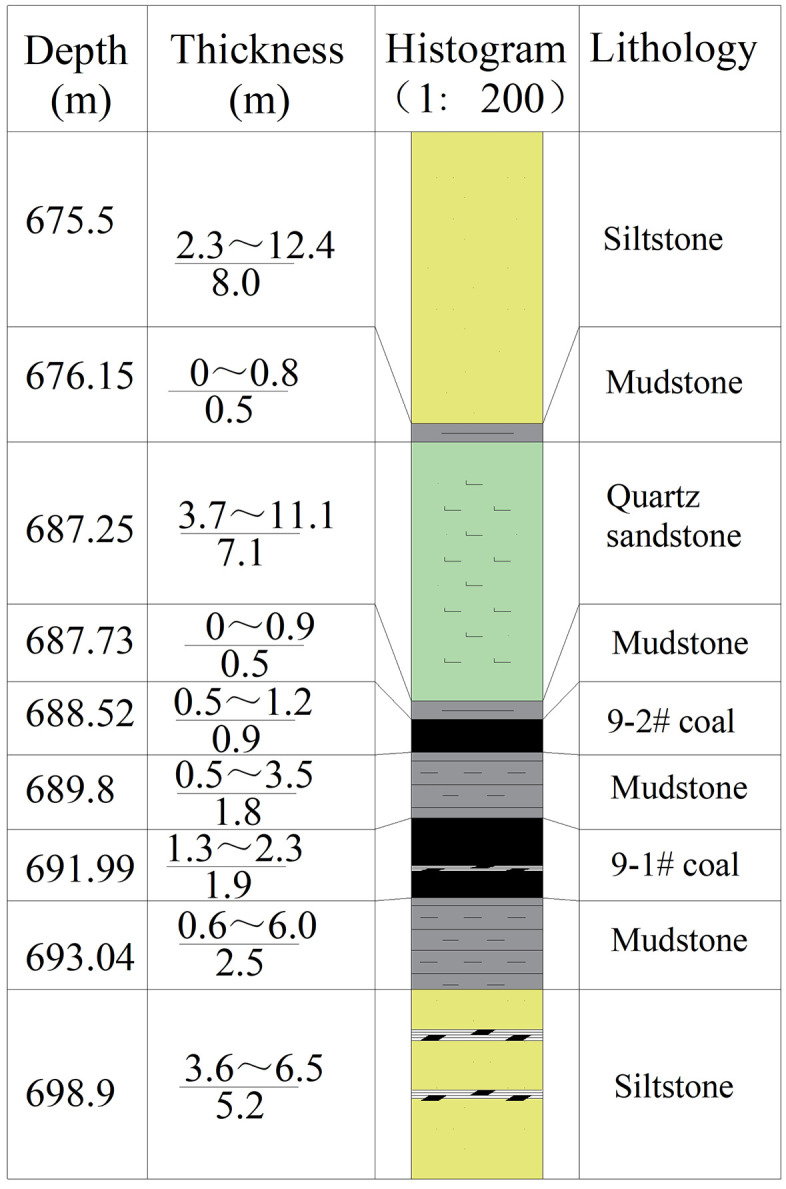
Geological Columnar Diagram of Coal and Rock Strata.

As shown in the bar chart of Fig 1, the coal seam 9 is overlain by a hard, rigidly overlying sandstone roof with a thickness ranging from 7.0 to 23.5 meters. This thick, rigid, overlying sandstone layer generates significant regional stress. Since the coal seam’s overall strength is far lower than that of the sandstone, the immense pressure from the overlying sandstone readily causes compaction and plastic deformation of the coal seam. This results in more severe fragmentation of the coal face and increased rib damage during longwall advancement, directly impacting safe and efficient coal recovery. The following challenges are anticipated during the recovery period of this face:

(1) The 7.0–23.5 m thick rigid, directly overlying sandstone roof caused excessively large initial pressure relief strides. Sudden roof failure poses a risk of secondary rock burst hazards.
(2) Fracturing of the directly overlying sandstone roof increases the periodic pressure relief stride during coal seam mining. Dynamic responses induced by mining activities cause intense rock pressure manifestations in the working face and both roadways during pressure periods, posing a risk of roof collapse.
(3) The coal body in the 11129 working face is affected by water from sandstone fractures in the roof, reducing its strength and increasing susceptibility to coal wall spalling and water inrushes in the working face.

## 3. Macroscopic fracture morphology and load-bearing characteristics of coal rock mass

### 3.1. Macroscopic fracture morphology analysis of coal rock mass

The physical model experiment is based on the engineering context of the 11129 working face at Zhangji Coal Mine. A steel frame with dimensions of 3.0 m (length) × 1.5 m (height) × 0.3 m (width) was employed to conduct physical experiments on the evolution of fractures in hard roof overburden and mining-induced instability. To simulate key characteristics of similar materials, experimental results should satisfy: geometric similarity, kinematic similarity, dynamic similarity, boundary condition similarity, and proportionality in corresponding physicochemical conditions. Similar materials such as sand, lime, and gypsum were mixed in appropriate ratios. Based on similarity theory, key parameters for the physical model experiment are designed as shown in Table 1 below.

**Table 1:** Basic parameters of physical modelling experiments.

Experiment name	Experimental parameters	Experiment name	Experimental parameters
Model length	300 cm	Time similarity ratio	1:12
Model width	30 cm	Excavation step length	5 cm
Model height	120 cm	Boundary dimensions	30 cm
Geometric similarity ratio	1:100	Excavation time interval	40 min
Bulk density similarity ratio	3:5	Total excavation duration	32 h
Stress similarity ratio	3:500	Upper load compensation	93.96 kPa

**Fig 2:**
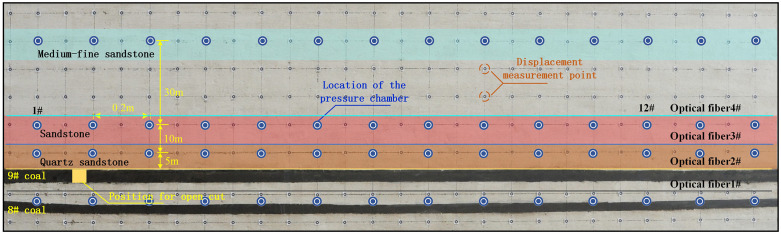
Fiber optic and displacement measurement point layout diagram.

As shown in Fig 2, three layers of YLH-500 k miniature soil pressure cells (range 0–500 kPa) are arranged above the 9 coal seam to monitor the deformation of the overburden at different stages (stable, separation, bending subsidence, and failure) during coal seam mining. Each row of pressure cells is numbered sequentially from left to right as #1 to #14. Pressure cells are arranged at equal intervals, with a spacing of 20 cm between cells within the same layer. Additionally, displacement measurement points are distributed at 10 cm intervals both horizontally and vertically across the model surface. The movement of surface displacement measurement points is utilized to monitor the degree of movement in the roof strata.

**Fig 3:**
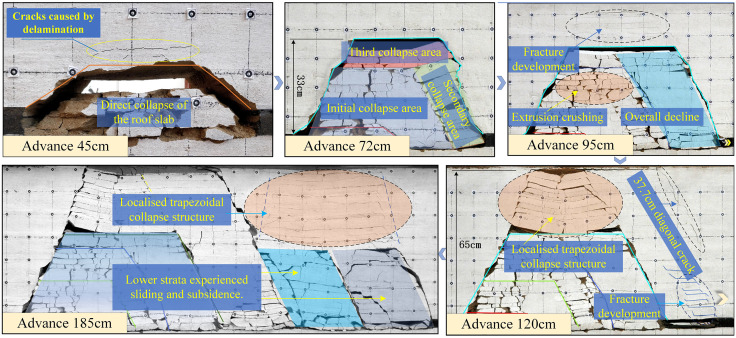
Similar experimental models reveal distinct structural evolution characteristics of coal and rock at varying distances.

First, establish the working face cut-off drift (30 cm from the left boundary of the model) and advance progressively to the right from the cut-off position. When the working face reaches 10–30 m, the roof exhibits a suspended state without reaching the ultimate bearing strength of the immediate roof. At 45 m, the immediate roof reached its bearing capacity limit, resulting in an initial fracture and collapse into the goaf, as shown in Fig 3. Following the immediate roof collapse, a trapezoidal space with a height of 7 m formed, using the goaf as its long side. Horizontal fissures developed in the basic roof above this space.

At the 72 m advance point, the second cyclic collapse occurred at a 12 m step interval. The overburden collapse formed a short cantilever structure, with the collapse zone height increasing to 33 m. After advancing another 10 m, a localized collapse occurred in the overburden approximately 5 m behind the coal face. The immediate roof and basic roof, primarily composed of sandstone, collapsed as a unified mass. The overlying softer rock types fractured into smaller blocks with layered separation.

The working face advanced another 20 m (reaching 95 m), causing the roof to fracture upward along the coal wall. Due to mutual support from the collapsed rock masses in the goaf, complete collapse did not occur at this stage, with the collapse height in the goaf reaching 35 m. Softer rock layers, influenced by their inherent primary fractures and weak planes, exhibited relatively higher fracture and fragmentation levels, forming irregular blocks after collapse. Within the 20-meter zone above the collapse zone, rock layers developed delamination fractures and underwent downward bending deformation. As the working face advanced another 5 meters (reaching 100 meters), the roof 5 meters behind the coal face subsided and collapsed relatively intact. The lower rock layers, subjected to gravitational compression from the overlying strata, exhibited a relatively higher number of fractures in the rock blocks after collapse.

The working face advanced another 25 m (reaching 120 m). Cracks formed in the medium-to-low strata, gradually causing them to delaminate, while the high strata developed a dipping shear fracture approximately 37.7 m in length. At this stage, the medium-to-fine sandstone bearing layer 45 m above the coal seam fractured, collapsing simultaneously with the overlying weak strata and locally forming a positive trapezoidal structure. After a 40-minute settling period, the lower immediate roof detached first and collapsed into the goaf, with block collapse extending approximately 20 m. The shear fracture developed downward to 40.3 m, followed by subsidence and collapse of the basic roof. The upper fracture length developed to 51.6 m.

The working face advanced to 185 m, with the last two cycles of pressure advance occurring at approximately 30 m intervals. The rock mass collapse patterns were similar, both starting from lower levels and gradually collapsing toward the middle strata. The collapsed blocks remained relatively intact, with a height of about 30 m. The overlying strata exhibited downward bending settlement in a positive trapezoidal shape. Influenced by the rock mass’s inherent primary joints and interfaces between different lithologies, the detachment joints predominantly exhibit a horizontal orientation.

**Fig 4:**
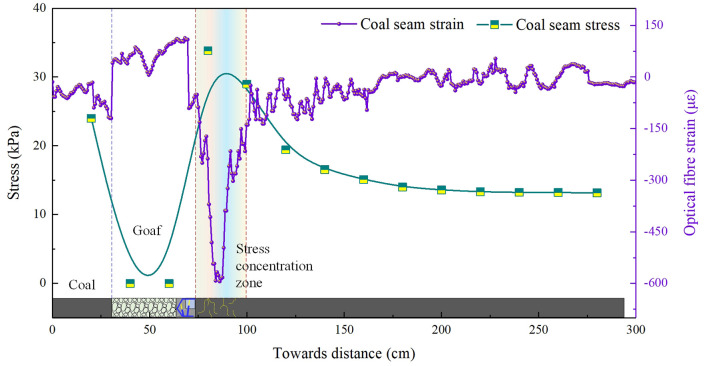
Evolutionary characteristics of coal-rock bearing capacity during 45 m of coal seam mining (model excavation: 45 cm).

To gain a more intuitive understanding of the load-bearing and stress characteristics of the coal body during longwall mining operations, data from the coal seam roof fibre optic sensor (Fibre 2#) and the pressure cells embedded within the coal seam were extracted for analysis. The stress-strain evolution patterns at different advance distances within the coal seam are illustrated in Fig 4.

The stress-strain curve for the 45-metre advance of the working face is shown in Fig 4. Stress concentration occurs 5–15 metres ahead of the coal face, with a peak stress value of 33.8 kPa. Stress values decay instantaneously at positions distant from the coal face. The peak strain occurs 11 metres behind the coal face, reaching a value of 594.3, before rapidly decreasing and levelling off. In the goaf behind the coal face, fibre optic data exhibited localised fluctuations due to the influence of fallen rock fragments, with overall values generally lower. As the pressure cells had been excavated during coal seam mining and no longer bore the load of fragmented rock masses in the goaf, all values recorded were zero.

**Fig 5:**
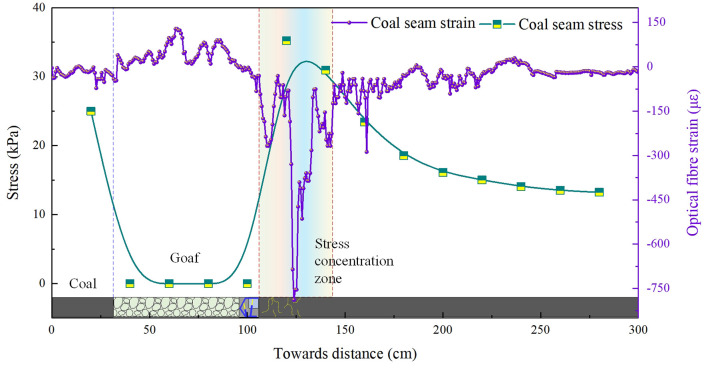
Evolutionary characteristics of coal-rock bearing capacity during 72 m of coal seam mining (model excavation: 72 cm).

**Fig 6:**
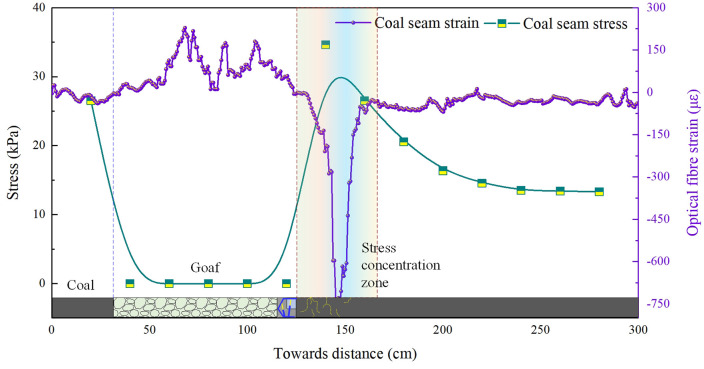
Evolutionary characteristics of coal-rock bearing capacity during 95 m of coal seam mining (model excavation depth: 95 cm).

As the working face advanced continuously to 72–95 m, the roof experienced two to three periodic fractures. The stress-strain evolution curves of the coal body are shown in Figs 5 and 6. Upon reaching 72 m, stress concentration occurred within 40 m ahead of the coal face, with peak stress of 35.2 kPa recorded at 18 m ahead, subsequently decaying to 13–15 kPa. Peak strain of 788.3 occurred at 21 m ahead, also diminishing thereafter. Upon advancing to 95 m, the stress and strain within the coal body exhibited minor fluctuations. The pattern and trend of the curves were broadly similar to previous fractures, though the numerical values differed.

**Fig 7:**
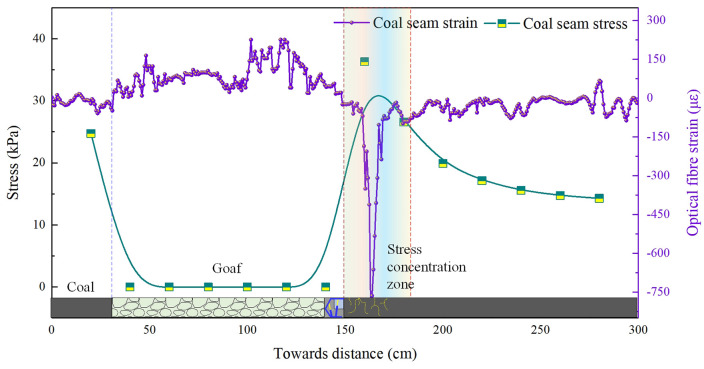
Evolutionary characteristics of coal-rock bearing capacity during 120 m of coal seam mining (model excavation depth: 120 cm).

**Fig 8:**
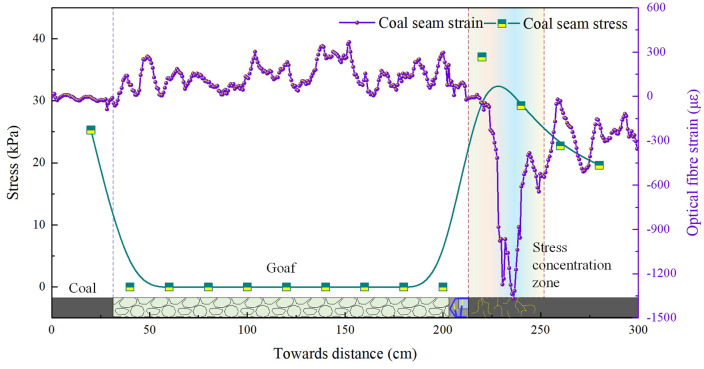
Evolutionary characteristics of coal-rock bearing capacity during 185 m of coal seam mining (model excavation depth: 185 cm).

The working face advanced continuously, with the roof undergoing multiple cyclic failures. Stress concentrations of varying degrees occurred on the solid coal body, as illustrated in Figs 7 and 8. As the working face advanced from 120 m to 185 m, stress-strain concentration zones formed within the coal body within 50 m of the advance face. Beyond this 50 m range, the stress on the coal body gradually diminished. Peak stress-strain values within the coal body occurred within the 5–20 m range ahead of the coal face. Overall peak pressure values fluctuated between 34.1 and 37.1 kPa, while peak fibre strain values ranged from 811.3 to 1367.5. A pressure relief zone formed in the goaf behind the coal face, where pressure sensor readings registered zero. Fibre-optic sensors deployed in the coal seam roof exhibited localised fluctuations due to roof collapse, with overall strain values fluctuating between 0 and 366.3.

### 3.2. Macroscopic stress field, displacement field, and energy field evolution characteristics of coal rock mass

#### 3.2.1. Establishment of numerical models.

**Fig 9:**
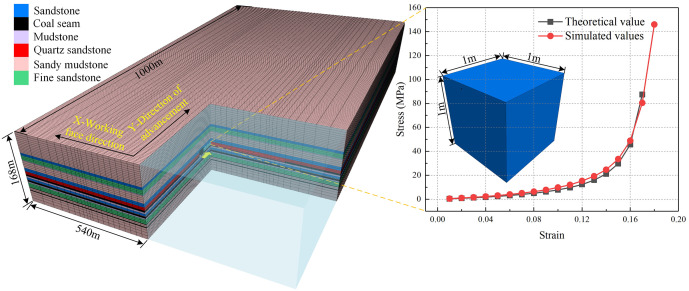
Three dimensional numerical model diagram.

A numerical model with dimensions length (540 m) × width (1000 m) × height (168 m) was established, as shown in Fig 9. The mechanical parameters of the roof and floor strata are listed in Table 2. During the mining process, the FLAC3D software could not fully simulate the collapse and backfilling of the goaf behind the working face. Numerous experts and scholars have conducted extensive experiments and research on the support effect of the equivalent backfilled rock mass in the goaf. The goaf was simulated using the Double-yield model, with backfill parameters listed in Table 3.

**Table 2:** Mechanical parameters of roof and floor rock strata at the coal seam.

Lithology	Density/ kg/m^3^	Bulk modulus /GPa	Shear modulus /GPa	Tensile strength/MPa	Cohesion /MPa	Internal friction angle/°
Sandy mudstone	2605	2.16	1.69	0.38	2.6	27
Siltstone	3211	10.48	8.19	0.44	2.85	28
Quartz sandstone	2600	21.03	13.53	0.47	2.8	30
9−2 # coal	1300	0.83	0.38	0.29	1.8	24
Mudstone	2554	3.23	1.85	0.32	2.2	28
9−1 # coal	1300	0.83	0.38	0.29	1.8	24
Mudstone	2554	3.23	1.85	0.32	2.2	28
Siltstone	3211	10.48	8.19	0.44	2.85	28
Sandy mudstone	2605	2.16	1.69	0.38	2.6	27
8 # coal	1300	0.83	0.38	0.29	1.8	24
Mudstone	2554	3.23	1.85	0.32	2.2	28

**Table 3:** Mechanical parameters of main rock materials in the goaf.

Category	Density /kg/m^3^	Bulk modulus /GPa	Shear modulus /GPa	Internal friction angle/°	Dilatancy angle/°
Value	2000	11.1	8.3	13	7

Based on the geological conditions of the field engineering case and the objectives of the simulation study, detailed simulation calculations were conducted. The specific simulation process is as follows:

(1) Constructed a numerical model based on the field engineering overview and simulation objectives, and assigned parameters to the simulated material medium while setting the initial stress field.
(2) Established boundary constraints for the model, completed stress equilibrium calculations for the original rock, and ensured the authenticity of the computational results.
(3) To mitigate boundary effects on the mining area simulation, a 200-meter coal pillar was established at the boundary of the 11129 working face, simulating the fully mechanized mining face from left to right.
(4) Simulation results were saved at key stages throughout the mining process. Distribution contour plots of mining-induced fracture patterns, stress fields, and displacement field evolution in the overburden were extracted for analysis.
(5) Utilized the embedded Fish language within the numerical simulation software to program energy calculations for model cells. By traversing model cells, generated distribution maps of accumulated mining energy to facilitate energy field evolution analysis. The formula used for model cell energy calculation is as follows.

Based on the definition of elastic energy density in energy principles, the accumulated elastic energy density *u*_*e*_ within a model medium cell under loading is:


$$u_{e}=\frac{\text{1}}{2E_{i}}\left[\sigma_{\text{1}}^{\text{2}}+\sigma_{\text{2}}^{\text{2}}+\sigma_{\text{3}}^{\text{2}}-2v_{i}\left(\sigma_{\text{1}}\sigma_{\text{2}}+\sigma_{\text{1}}\sigma_{\text{3}}+\sigma_{\text{2}}\sigma_{\text{3}}\right)\right]$$ (1)

Where: *E*_*i*_—Elastic modulus of the model medium unit cell, GPa. *v*_*i*_—Poisson’s ratio of the model medium unit cell. *σ*_*1*_, *σ*_*2*_, *σ*_*3*_—Maximum, intermediate, and minimum principal stresses of the model medium unit cell, MPa.

#### 3.2.2. Analysis of numerical results.

**Fig 10:**
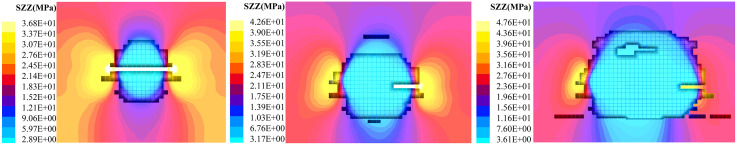
Stabilization evolution of the mining stress field of coal rock in hard-roof condition quarries (50 ~ 90 m stage of working face mining back). (a) Working face advanced 50 m. (b) Working face advanced 70 m. (c) Working face advanced 90 m.

**Fig 11:**
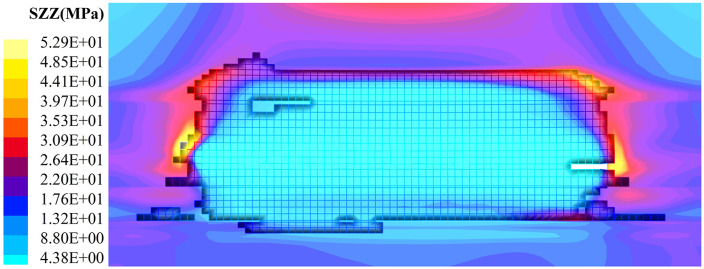
Stabilization evolution of the mining stress field of coal rock in hard-roof condition quarries (240 m stage of working face mining back).

Extract stress contour maps from different mining phases of the model to analyze the evolution of load-bearing characteristics in coal and rock bodies. To visually represent stress trends at various locations within the mining area, cross-sectional slices are made along the mid-strike and mid-dip directions. Simultaneously, slices parallel to the working face are made along the coal seam floor. The results are shown in Figs 10–11.

The roof experienced an initial collapse when the working face advanced to the 50 m position (including the cut-off area), as shown in Fig 10(a). An area of advanced and lateral stress concentration formed in front of the coal walls surrounding the goaf, with a peak stress of 33.1 MPa and a stress concentration factor of 1.7. Stress gradually recovered to the original rock stress as distance from the working face increased. The goaf served as a stress-relief zone, with the overlying plastic zone (white grid lines) indicating shear or tensile failure of the coal-rock mass under stress. Overall stress values remain below those of the original rock mass. Lower-level caving zones act as backfill for the goaf, thereby reducing stress transmission from overlying loads. As the working face advances to 70–90 m, as shown in Fig 10(b) and (c), periodic caving occurs following the initial roof failure and subsequent mining operations. The peak advance stress generated by the solid coal surrounding the working face increases. At 70 m advance, peak stress rises to 38.8 MPa. As the extent and height of the goaf collapse increase, the plastic zone expands. At 90 m advance, peak stress reaches 42.3 MPa, with a stress concentration factor of 2.2.

The continuous advancement of the working face further expanded the goaf space. Influenced by mining strides and backfill materials, the goaf exhibited undulating changes resembling waves. Overall stress levels within the goaf generally remained lower than those of the original rock mass. This indicates that during this phase (240 m), the goaf experienced significant overall stress relief, though the degree of stress reduction was slightly lower compared to previous mining cycles. The overlying strata formed an arched stress relief zone, indicating that this area had lost its bearing capacity and had undergone fracturing or deformation. The development height of the plastic zone near the coal face was higher than that in the central part of the goaf. At the 240 m advance position, the peak stress at the coal face increased to 52.9 MPa, with a stress concentration factor of 2.78.

**Fig 12:**
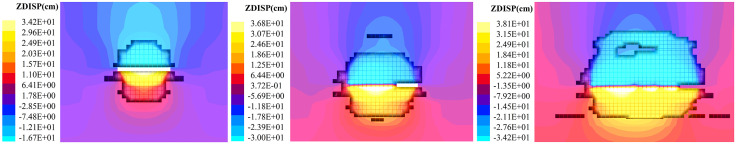
Settlement characteristics of coal rock mining displacement field in hard roof condition quarries (50 ~ 90 m stage of working face mining back). (a) Working face advanced 50 m. (b) Working face advanced 70 m. (c) Working face advanced 90 m.

**Fig 13:**
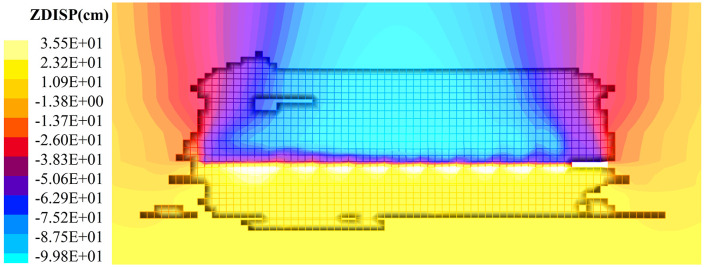
Settlement characteristics of coal rock mining displacement field in hard roof condition quarries (240 m stage of working face mining back). **(a)** Working face advanced 190. **(b)** Working face advanced 240 m.

Displacement changes in the overburden during coal seam mining also reflect the degree of movement in the coal-rock strata. Figs 12 to 13 shows the fracture settlement contour maps of the coal-rock mass at different mining stages.

As the working face advances 50–90 m, goaf spaces of varying sizes form behind the coal wall. Within the goaf, the coal seam floor undergoes upward expansion deformation, resulting in positive values, while the overlying strata above the coal seam exhibit negative values due to fracturing and subsidence. The initial subsidence of overlying strata following the first fracturing (after 50 m advance) ranges from 7.5 to 16.7 cm. The collapse patterns of strata at different levels show an evolution trend toward multi-layered semi-circular arches. The coal body in front of the longwall faces undergoes compressive deformation due to the influence of the advance stress concentration zone, with compression ranging from 2.8 to 12.1 cm within the stress concentration area. As the mining distance along the strike increases, the subsidence of the overburden within the goaf gradually increases, with the vertical displacement of the collapse zone rising from 16.7 cm to 34.2 cm.

As the working face continues to advance, the overall subsidence of the coal and rock strata steadily increases. The floor of the goaf exhibits upward uplift, though the magnitude of this uplift diminishes with increasing distance from the working face. This occurs because the extended strike distance causes fracturing and subsidence of the overlying strata at medium-to-high levels into the goaf. Within the goaf, vertical stress intensifies, gradually compacting the rock masses in the collapse zone. The contour map indicates increased subsidence in the central part of the goaf, thereby inhibiting the development of floor uplift. The increasing strike distance of mining causes successive fracturing and instability in the coal and rock strata at medium to high levels, increasing the stress on the coal and rock strata adjacent to the solid coal body. The contour map shows a gradual increase in the degree of compression of the coal and rock strata.

As shown in Fig 13, when the working face advanced to 240 m, the maximum subsidence of overlying strata in the goaf ranged from 82.2 to 99.8 cm, while the maximum floor uplift decreased from 37.3 cm to 35.5 cm. Displacement gradually diminished with increasing distance from the goaf. At this stage, the solid coal exhibits varying degrees of compression at different locations under the influence of the advance stress concentration zone, with deformation ranging from 13.7 to 87.5 cm.

**Fig 14:**
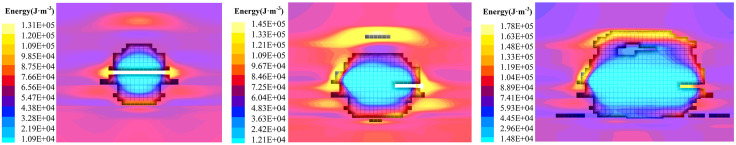
Characteristics of the evolution of the energy field of coal rock mining in hard-roof condition quarries (50 ~ 90 m stage of working face mining back). (a) Working face advanced 50 m. (b) Working face advanced 70 m. (c) Working face advanced 90 m.

**Fig 15:**
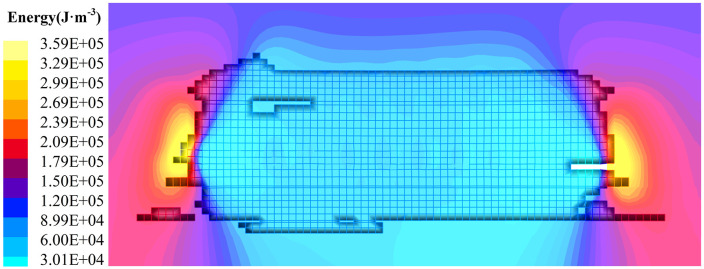
Characteristics of the evolution of the energy field of coal rock mining in hard-roof condition quarries (240 m stage of working face mining back).

Coal and rock masses undergo energy accumulation and dissipation during load-bearing or fracture moments. This energy behavior effectively reflects the load-bearing characteristics and damage levels of coal and rock strata. The goaf behind the coal face is filled with coal and rock blocks located within the collapse zone. These fill blocks experience minimal influence from loads transmitted by overlying strata, manifesting as low energy values. The uncollapsed, hard rock layers above bear their own weight and that of the softer overlying strata, exhibiting significant energy accumulation reflected as large numerical values. Details of energy accumulation in coal and rock strata during different stages of coal seam mining are shown in Figs 14–15.

The initial roof collapse occurred at 50 m into coal seam mining, as shown in Fig 14(a). As the span length of the hard rock strata increased, the accumulated energy rose. When the working face advanced 50 m, the roof fractured and collapsed, transferring stress to the goaf and releasing energy. Post-release energy fluctuated within the range of 11.3–41.9 kJ/m³. Energy accumulates within the overlying strata above the goaf, with values ranging from 62.3 to 103 kJ/m³. At this stage, the solid coal-rock mass surrounding the goaf undergoes compressive deformation due to concentrated advance or lateral stresses, leading to internal energy accumulation. The maximum accumulated energy reaches 124 kJ/m³. As the working face advances to 70 m and 90 m, as shown in Fig 14(b) and (c), the increased strike distance of the goaf leads to both greater energy accumulation and a broader range within the overlying bearing layer. Simultaneously, the degree of energy accumulation within the solid coal seam also increases. This indirectly indicates that the damage to the coal-rock strata intensifies with the accumulation of energy. The maximum energy accumulation in the coal-rock strata at 70 m and 90 m advance reached 133 kJ/m³ and 149 kJ/m³, respectively.

The working face advances continuously, causing the caving zone to fracture and subside toward higher strata, moving away from the goaf. The fracture morphology of the overburden exhibits an inverted trapezoidal shape with a shorter upper base and longer lower base. As the unsupported span gradually decreases, the accumulated energy within the bearing strata diminishes compared to lower strata. The expansion of the goaf space formed multiple inverted-step cantilever structures above the solid coal near the working face, exerting forces onto the solid coal-rock strata. The load-bearing stress on the coal-rock strata increased, and the accumulated energy within them also continued to grow. As the working face advanced 240 m, the energy accumulation density in the overlying strata above the goaf decreased from 99.7 kJ/m³ to 81.3 kJ/m³. The energy accumulation in the solid coal mass ahead of the coal wall increased from 298 kJ/m³ to 242 kJ/m³.

## 4. Microscopic damage characteristics of coal rock mass

### 4.1. Experimental system introduction

**Fig 16:**
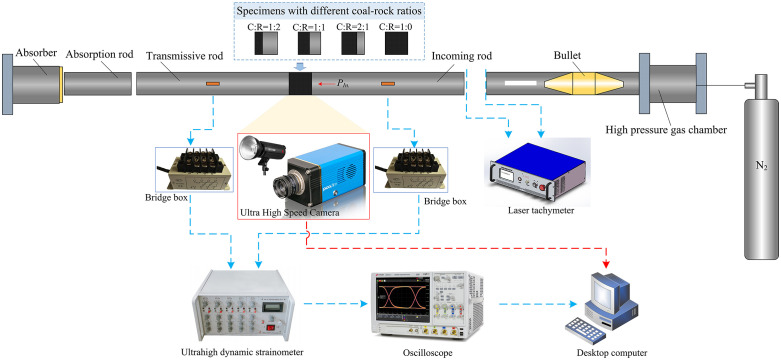
SHPB test set system.

A laboratory-scale, modified 50 mm-diameter separated Hopkinson bar system was employed. The schematic diagram of the test system setup is shown in Fig 16, with the test apparatus parameters listed in Table 4. The test system comprises a velocity measurement system, a bar system, a data acquisition system, a loading system, and a high-speed camera system.

**Table 4:** Parameters of the SHPB device.

Name	Length/mm	Calibre/mm	Type of material	Elastic modulus/GPa	Poisson’s ratio	Density/kg·m^-3^	Wave speed/m·s^-1^
Incoming rod	2000	50	40Cr alloy steel	210	0.3	7800	5180
Transmissive rod	1500						
Absorption rod	500						

Rock cores retrieved from the coal-rock composite at the site were cored, cut, and polished into specimens meeting SHPB test specifications. Four specimens with different coal-to-rock ratios were prepared and subjected to water saturation treatment. During the working face mining process, the roof underwent three stages: subsidence deformation, initial caving, and cyclic caving. To simulate the impact of the roof on the coal body during different stages, and considering the impact results of several samples, current research experience, literature data, and laboratory equipment limitations, coal impact tests were conducted at pressures of 0.3 MPa, 0.5 MPa, and 0.7 MPa. Each group of coal samples underwent three repeated impacts, with the most effective test results selected for analysis. The test design is shown in Table 5.

**Table 5:** Impact scheme of test coal samples.

Impact strength/MPa	Coal-rock ratio	Specimen number	Calibre/mm	Height/mm
0.3	C:R = 1:0	C:R = 1:0 (1)	50.14	50.24
		C:R = 1:0 (2)	51.12	50.88
		C:R = 1:0 (3)	49.76	50.13
	C:R = 2:1	C:R = 2:1 (1)	51.06	51.18
		C:R = 2:1 (2)	51.31	50.24
		C:R = 2:1 (3)	51.21	50.20
	C:R = 1:1	C:R = 1:1 (1)	50.22	49.87
		C:R = 1:1 (2)	49.99	50.04
		C:R = 1:1 (3)	49.87	50.15
	C:R = 1:2	C:R = 1:2 (1)	50.46	50.21
		C:R = 1:2 (2)	50.21	49.88
		C:R = 1:2 (3)	51.01	51.01
0.5	C:R = 1:0	C:R = 1:0 (1)	49.91	50.02
		C:R = 1:0 (2)	50.32	50.07
		C:R = 1:0 (3)	49.91	49.95
	C:R = 2:1	C:R = 2:1 (1)	49.99	50.97
		C:R = 2:1 (2)	51.03	51.05
		C:R = 2:1 (3)	50.02	50.38
	C:R = 1:1	C:R = 1:1 (1)	50.26	51.12
		C:R = 1:1 (2)	51.23	50.22
		C:R = 1:1 (3)	49.98	51.11
	C:R = 1:2	C:R = 1:2 (1)	50.07	50.01
		C:R = 1:2 (2)	50.22	49.79
		C:R = 1:2 (3)	50.02	50.13
0.7	C:R = 1:0	C:R = 1:0 (1)	50.12	51.14
		C:R = 1:0 (2)	50.30	50.54
		C:R = 1:0 (3)	49.87	50.18
	C:R = 2:1	C:R = 2:1 (1)	50.06	50.18
		C:R = 2:1 (2)	50.21	51.22
		C:R = 2:1 (3)	49.96	50.08
	C:R = 1:1	C:R = 1:1 (1)	50.13	49.96
		C:R = 1:1 (2)	49.99	50.02
		C:R = 1:1 (3)	49.87	51.08
	C:R = 1:2	C:R = 1:2 (1)	50.16	50.34
		C:R = 1:2 (2)	50.11	49.97
		C:R = 1:2 (3)	50.02	51.01

### 4.2. Micro-scale progressive damage characteristics of coal rock mass

**Fig 17:**
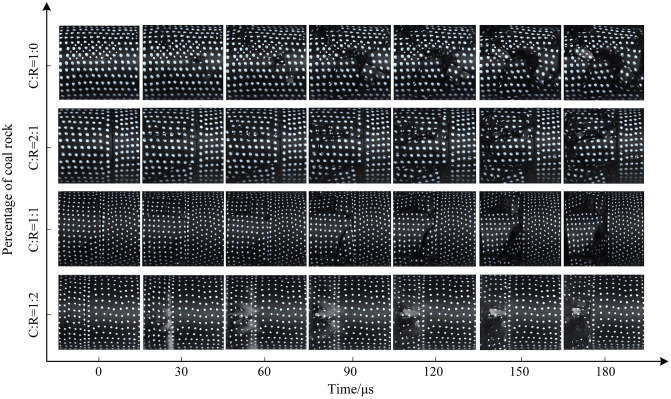
Surface crack evolution characteristics of coal-rock composite at 0.3 MPa gas pressure.

**Fig 18:**
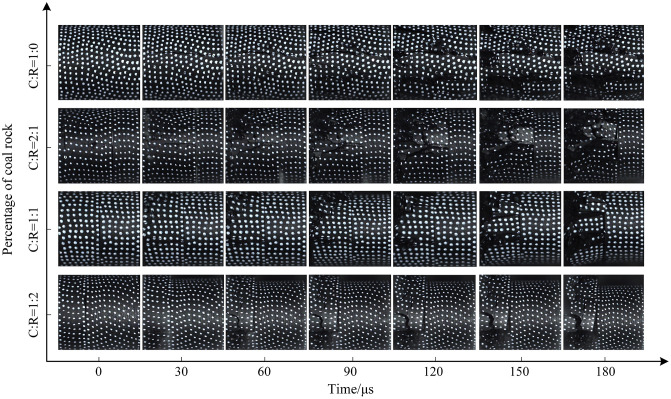
Surface crack evolution characteristics of coal-rock composite at 0.5 MPa gas pressure.

**Fig 19:**
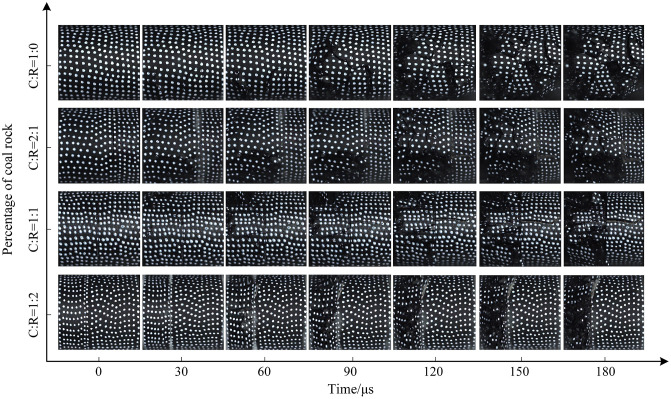
Surface crack evolution characteristics of coal-rock composite at 0.7 MPa gas pressure.

The macroscopic crack propagation patterns in coal rock are crucial for studying the strength, stability, and failure behavior of coal bodies. First, we marked white dots on the coal sample surface using a white marker pen (to prepare for subsequent surface speckle pattern recognition). The crack evolution characteristics of saturated water-coal rock composites under different impact pressures are shown in Fig 17–19 below. Under dynamic impact loading, cracks initiate locally within the coal-rock body, progressively develop and propagate, ultimately leading to complete specimen failure—a continuous evolutionary process. In the water-saturated state, elevated internal moisture content softens the coal-rock matrix. The specimen ends did not exhibit significant crushing, resulting in slower crack initiation and propagation. The specimen demonstrated overall slow failure characteristics.

Applying 0.3 MPa gas pressure, the progressive damage characteristics of the coal-rock body are shown in Fig 17. When the coal-to-rock ratio is 1:0, radial cracks appear in the central region of the coal sample at 30μs, gradually extending toward the diagonal position. As the stress wave propagates, the crack propagation direction forms a 45° angle with the horizontal plane. At 180μs, the overall fragmentation of the coal sample was relatively coarse, with a relatively small number of cracks. When the coal-to-rock ratio was 2:1 (C:R = 2:1), a diagonal shear crack with an inclination of approximately 30° developed in the lower part of the coal sample at 30μs, while a horizontal crack simultaneously formed in the central region. At 90μs, numerous fine cracks extended along the horizontal fracture line. As the fractures developed, the coal portion of the specimen gradually flaked off, ultimately leading to unstable fracture. The rock portion remained relatively intact without significant cracking. At a coal-to-rock ratio of 1:1 (C:R = 1:1), a horizontal fracture first formed, followed by multiple vertical fractures. These cracks developed, interconnected, and ultimately caused failure of the coal sample section. When the rock proportion increased to C:R = 1:2, compression failure first occurred at the coal-rock interface, followed by outward spalling. Due to the lower proportion of coal and its overall lower strength compared to rock, the coal sample section exhibited a higher degree of damage.

The impact strength increased to 0.5 MPa. Pure coal specimens developed two primary cracks along the horizontal direction between 30 μs and 60 μs, with accelerated crack propagation and increased fragmentation of the coal sample. Details of specimen crack evolution are shown in Fig 18. At a coal-to-rock ratio of 2:1 (C:R = 2:1), upper coal flaking initiated at 60μs, with cracks propagating toward the specimen center. By 120μs, localized coal bulging occurred, followed by fragmentation into irregular blocks elsewhere, while the rock fraction retained relatively intact integrity. With equal coal-to-rock ratios, cracks initiated at the left end of the specimen at 30μs, leading to gradual instability and failure. As the rock proportion increased (C:R = 1:2), cracks primarily developed parallel to the impact gas pressure direction, causing upward slippage of the coal sample. The rock portion remained relatively intact, as the 0.5MPa impact strength was insufficient to reach its dynamic ultimate compressive strength.

The dynamic load intensity was increased to 0.7 MPa, and the crack evolution characteristics are shown in Fig 19. Compared to medium and low gas pressures, cracks appeared more rapidly and with greater density. The patterns of crack development and penetration were similar, but the increased impact gas pressure resulted in more severe fragmentation of the coal sample. At 30μs, multiple horizontal and vertical cracks appeared at the lower end of the pure coal specimen. By 90μs, cracks gradually developed toward the center of the coal sample, with localized spalling occurring. At a coal-to-rock ratio of 2:1 (C:R = 2:1), the high dynamic load intensity caused fine cracks to form first near the rock interface at the lower part of the coal sample. Over time, debris fragments detached around these cracks. Due to the high dynamic load strength, a primary crack developed horizontally within the rock matrix. At a coal-to-rock ratio of 1:1 (C:R = 1:1), the crack development pattern was fundamentally similar to that observed at 0.5 MPa. However, the higher pressure resulted in greater fragmentation of the coal sample and the formation of horizontal cracks within the rock. At a coal-to-rock ratio of 1:2 (C:R = 1:2), the low strength and thin thickness of the coal sample caused it to be partially crushed directly under high dynamic impact, exhibiting a higher degree of fragmentation compared to medium and low pressures. The rock sample remained largely intact, primarily due to its greater thickness, which made it less susceptible to reaching its dynamic ultimate compressive strength.

After coal samples become saturated with water, increased moisture content causes changes in the physical properties of the specimen, specifically softening, thereby reducing its impact resistance. Increased impact pressure accelerates crack propagation, penetration, and failure within the sample, rarely forming larger intact blocks. A higher proportion of rock within the sample reduces its thickness and hastens fragmentation. This occurs because rock possesses significantly higher density and hardness than coal. An increased rock content enhances energy transmission capacity while reducing energy absorption, and it has not yet reached its dynamic ultimate compressive strength. Greater energy impacts on the coal sample intensify its fragmentation.

### 4.3. Microscopic deformation and strain evolution characteristics of coal rock mass

**Fig 20:**
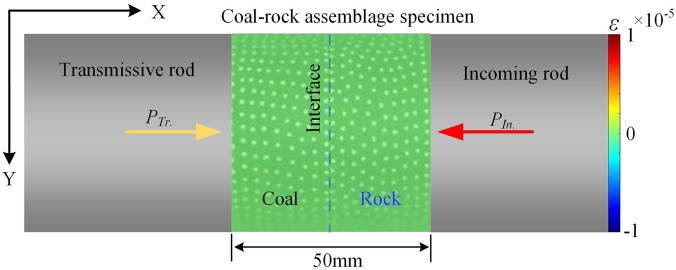
Initial state calibration diagram for coal samples.

To facilitate the extraction of surface values from coal samples, a coordinate reference system is established. The radial direction (impact gas pressure direction) is defined as the x-axis, and the vertical direction as the y-axis, as shown in Fig 20.

**Fig 21:**
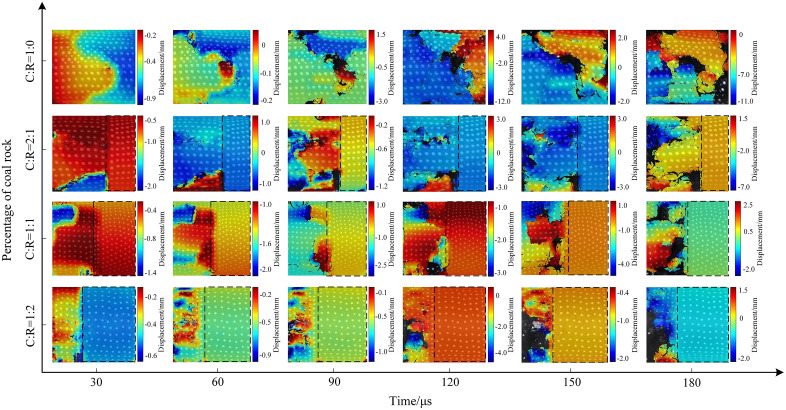
Surface displacement contour map of coal-rock complex at 0.3 MPa gas pressure.

**Fig 22:**
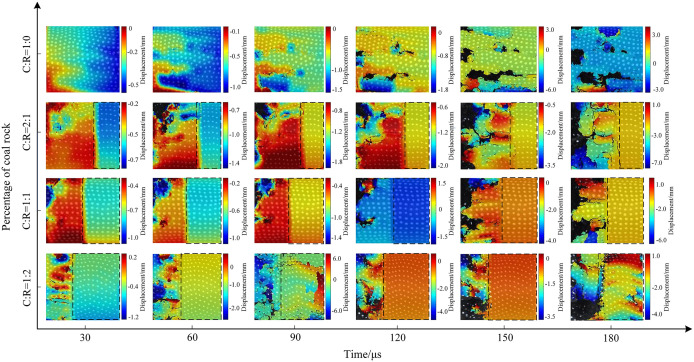
Surface displacement contour map of coal-rock complex at 0.5 MPa gas pressure.

**Fig 23:**
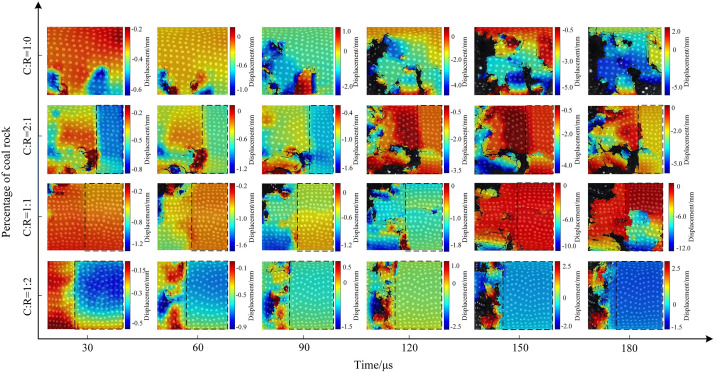
Surface displacement contour map of coal-rock complex at 0.7 MPa gas pressure.

Using MATLAB processing software, extract the motion trajectories of speckles on the coal sample surface. Take the initial speckle pattern of the specimen during impact loading as a reference, and select speckle patterns at typical moments during the loading process as deformation images for analyzing the experimental speckle images. Figs 21–23 show the displacement change contour plots for specimens subjected to different impact pressures.

The maximum displacement of coal-rock composites under different impact intensities often occurs near cracks. When the incident wave acts on the specimen’s end face, it undergoes a compression process in the horizontal direction. The presence of internal microcracks and weak planes causes localized slip instability, leading to the initial crack formation and failure. Fig 21 shows the displacement contour map of the specimen under 0.3 MPa impact pressure. In the pure coal specimen, surface zoning appeared at 30 μs, with significant displacement variations within the specimen. At 60 μs, the maximum displacement shifted along the crack propagation path, and as the crack width increased, the surrounding displacement gradually grew. At a coal-to-rock ratio of 2:1 (C:R = 2:1), the rock’s high integrity prevented failure, resulting in negligible surface displacement changes. The coal portion exhibited an initial displacement of 2 mm, increasing to a maximum of 7 mm as cracks propagated, penetrated, and caused specimen fragmentation. The rock portion fluctuated within a 1.5–2 mm range due to vibration effects. At a coal-to-rock ratio of C:R = 2:1, the rock portion showed negligible changes. The coal sample reached a maximum displacement of 4 mm at 150 μs before exfoliation occurred. At C:R = 1:2, multiple regions on the coal sample surface exhibited varying degrees of deformation, with the specimen reaching a maximum displacement of 4 mm at 90 μs.

The impact strength increased to 0.5 MPa, with the specimen displacement contour plot shown in Fig 29. The length and width of surface cracks on the specimens increased, with overall displacement variations rising. During the initial stage (30μs), the stress wave from the pure coal specimen (C:R = 1:0) acted directly on the rock end face, resulting in more pronounced displacement changes near the impact side. The maximum displacement ranged from 0.5 to 1 mm. Subsequently, the coal specimen fractured and exfoliated, with the final displacement peak reaching 3 mm. For C:R = 2:1, rock displacement increased from 0.7 mm to 3 mm between 30 μs and 180 μs. The coal portion exhibited more pronounced deformation, with a maximum displacement of 0.6 mm at 30 μs and 7 mm at 180 μs, resulting in a maximum surface displacement difference of 8 mm. As the rock proportion increased, localized spalling occurred in the coal portion.

The evolution trend of the surface displacement contour map of the specimen under an impact pressure of 0.7 MPa is shown in Fig 30. The increase in pressure further intensified the amplitude of movement after specimen fracture. The overall surface displacement in the pure coal state was significantly higher than that of pure coal specimens subjected to impact pressures of 0.3 MPa and 0.5 MPa. At 180 μs, the maximum surface displacement difference of the coal specimen reached 7 mm. At C:R = 2:1, high impact pressure caused horizontal cracks in the rock fraction, which connected and penetrated the coal sample, ultimately leading to overall instability and failure of the specimen. As the proportion of coal samples decreased, the degree of fragmentation increased. Due to the compressive forces between the fragmented particles, the displacement of the fragmented particles was relatively small, with the maximum displacement difference between fragmented particles being 4 mm.

The strain contour plots of the specimens further elucidate the failure mechanism and deformation characteristics of coal samples. In engineering practice, coal bodies subjected to factors such as strata loading, surrounding rock pressure, and water pressure undergo changes in their internal stress state. The evolution of strain provides valuable insights into the trends of stress application and deformation within coal bodies.

**Fig 24:**
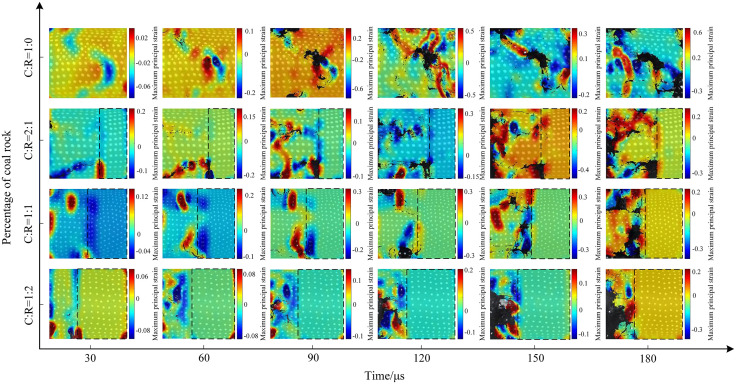
Contour map of maximum principal strain on the surface of the coal-rock composite at 0.3 MPa gas pressure.

The evolution of the maximum principal strain in specimens all originates from failure at a specific point within the coal sample. Over time, this failure propagates and connects to form multiple cracks, expanding along both sides of the cracks. The evolution of the maximum principal strain under 0.3 MPa impact pressure is shown in Fig 24. For pure coal samples, the initial cracking phase (30–60 μs) produced strain along vertical microcracks. Between 90 and 180 μs, cracks gradually developed and extended, with the maximum principal strain fluctuating between 0.2 and 0.6 during this evolution. At a coal-to-rock ratio of 2:1 (C:R = 2:1), strain values remained below 0.2 prior to 90 μs. The maximum strain value of 0.3 occurs at the crack propagation location at 180 μs. As the rock proportion increases to C:R = 1:1, the maximum principal strain difference at the interface reaches 0.5 at 90 μs. The evolution process resembles that of C:R = 1:1, with reduced coal sample thickness diminishing dynamic impact resistance.

**Fig 25:**
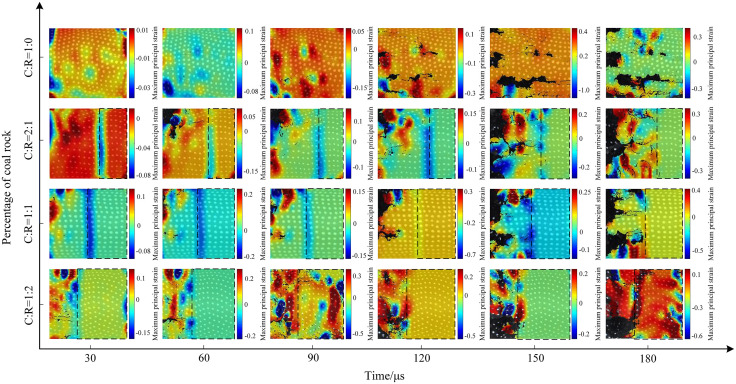
Contour map of maximum principal strain on the surface of the coal-rock composite at 0.5 MPa gas pressure.

**Fig 26:**
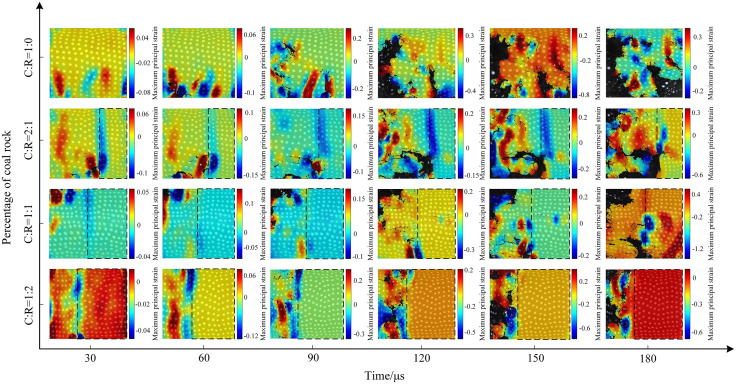
Contour map of maximum principal strain on the surface of the coal-rock composite at 0.7 MPa gas pressure.

The evolution process of the maximum principal strain cloud under 0.5 MPa impact pressure is shown in Fig 25. At 150 μs, the maximum principal strain value fluctuates between −0.3 and 0.3 for a C:R = 2:1 ratio, decreasing as the proportion of coal sample decreases. At a C:R ratio of 1:2, local flaking of the coal sample occurred at 180 μs, resulting in surface strain values ranging from −0.6 to 0.3. When the pressure increased to 0.7 MPa, as shown in Fig 26, the degree of specimen fragmentation further intensified. At a C:R ratio of 1:1, the maximum strain value increased to 1.2, primarily concentrated at the interface of the coal-rock composite.

**Fig 27:**
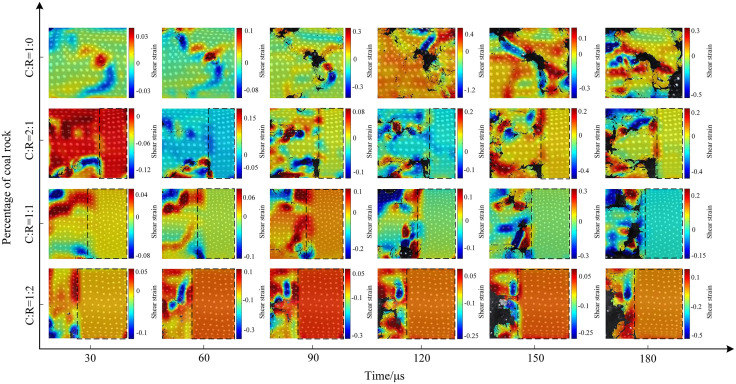
Surface shear strain contour map of coal-rock composite at 0.3 MPa gas pressure.

**Fig 28:**
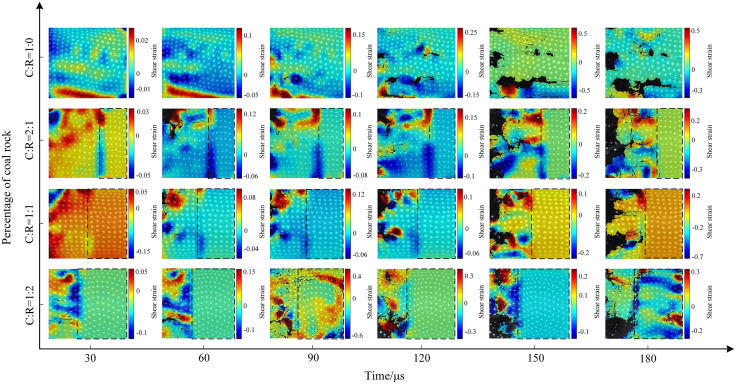
Surface shear strain contour map of coal-rock composite at 0.5 MPa gas pressure.

**Fig 29:**
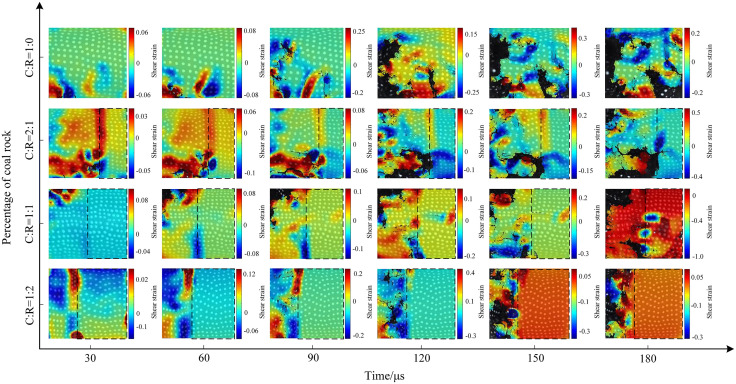
Surface shear strain contour map of coal-rock composite at 0.7 MPa gas pressure.

The evolution patterns of surface shear strain for coal-rock mixtures with varying water saturation ratios are shown in Figs 27–29. Due to factors such as the water saturation properties and pore structure of the coal-rock, the trends in shear strain vary with different impact air pressures, with shear strain gradually increasing as the impact air pressure rises. At a coal-to-rock ratio of 1:1 (C:R = 1:1), during the gradual increase of impact pressure from 0.3 MPa to 0.5 MPa and 0.7 MPa, the maximum shear strain values during the fragmentation stage (180 μs) were 0.2, 0.7, and 1.0, respectively. At high pressures, the shear strain variation characteristics of the specimens become more pronounced. As the rock proportion increases, the maximum shear strain gradually shifts toward the coal sample section, resulting in varying degrees of shear failure. Since the rock portion remains largely undamaged, its shear strain variation is not significant.

The propagation mechanism of stress waves in SHPB tests must first establish two fundamental assumptions: one-dimensional stress waves and homogeneity. By converting the voltage signal from an oscilloscope into strain and applying the principle of stress wave reflection, the relationships between material stress, strain, strain rate, and stress waves are derived [21]:


$$\left\{ \begin{array}{c} @l@\sigma \left(t\right)=\frac{EA}{A_{S}}\varepsilon_{T}\left(t\right)\\ \varepsilon \left(t\right)=-\frac{2C}{l_{S}}\int_{\text{0}}^{t}\varepsilon_{R}\left(t\right)dt\\ \mathrm{\backslash stackrel}⬝\varepsilon \left(t\right)=-\frac{2C}{l_{S}}\varepsilon_{R}\left(t\right) \end{array}\right.$$ (2)

where: *ε*_*R*_*(t)*, *ε*_*T*_*(t)*—represent the strain in the rod corresponding to the reflected wave and transmitted wave propagating independently at time t, respectively. *A*, *E*, *C*—denote the cross-sectional area (m²), elastic modulus (MPa), and longitudinal wave velocity (m/s) of the elastic compression rod, respectively. *A*_*S*_, *l*_*S*_—represent the specimen cross-sectional area (m²) and original length (m), respectively.

The dynamic compressive strength of coal-rock composites is related to their internal structure and composition. Upon impact, internal microscopic pores undergo compression, leading to deformation and failure of the coal sample. The deformation characteristics and degree of fragmentation of the specimen are determined by the impact velocity. At lower impact velocities, the coal sample can fully absorb the impact energy without significant deformation. At higher impact velocities, the stress-strain curve of the coal sample under dynamic loading exhibits almost no compaction phase, absorbing less impact energy. Following the stress peak, the sample enters an unloading phase due to the release of elastic strain energy, accompanied by irreversible plastic deformation. Higher impact velocities subject the coal sample to greater pressure, indirectly enhancing its dynamic compressive strength.

**Fig 30:**
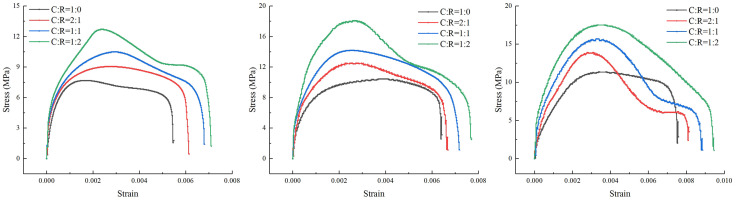
Dynamic stress-strain curves of coal-rock complexes under different impact pressures. (a) 0.3MPa. (b) 0.5 MPa. (c) 0.7 MPa.

The stress-strain curves of saturated coal-rock composites under different impact pressures are shown in Fig 30. The dynamic compressive strength of the specimens increased to a certain extent with rising impact pressure, accompanied by a corresponding increase in strain. At the low pressure of 0.3 MPa, the dynamic compressive strength of pure coal specimens was the lowest, peaking during the early compression phase before gradually decaying, with a peak dynamic compressive strength of 7.67 MPa. As the rock content in the specimens increased, the overall dynamic compressive strength of the specimens also gradually increased. With the rise in impact pressure, both the overall dynamic compressive strength and strain of the specimens increased. The overall trend of the stress-strain curves was fundamentally similar, exhibiting an arched pattern throughout the process. When the impact pressure reached 0.7 MPa, the peak dynamic compressive strengths for C:R = 1:0, C:R = 2:1, C:R = 1:1, and C:R = 1:2 were 11.36 MPa, 13.9 MPa, 15.6 MPa, and 17.52 MPa, respectively.

### 4.4. Microscopic energy evolution characteristics of coal rock mass

The voltage signals from the incident rod and transmission rod are collected via a hyperdynamic strain gauge. Based on one-dimensional stress wave theory, the electrical signals are converted into strain. The incident wave, reflected wave, and transmitted wave carry incident energy *W*_*I*_, reflected energy *W*_*R*_, and transmitted energy *W*_*T*_, respectively. The energy WS absorbed and dissipated during specimen failure under impact causes micro- or macro-level damage to the specimen. The calculation formula is [22]:


$$\left\{ \begin{array}{c} @l@W_{I}\left(t\right)=AEC\int_{\text{0}}^{t}\varepsilon_{I}^{\text{2}}\left(t\right)dt\\ W_{R}\left(t\right)=AEC\int_{\text{0}}^{t}\varepsilon_{R}^{\text{2}}\left(t\right)dt\\ W_{T}\left(t\right)=AEC\int_{\text{0}}^{t}\varepsilon_{T}^{\text{2}}\left(t\right)dt \end{array}\right.$$ (3)

Where: *ε*_*I*_*(t)*, *ε*_*R*_*(t)*, *ε*_*T*_*(t)*—represent the incident strain, reflected strain, and transmitted strain at time t, respectively.

Neglecting energy losses due to friction between the elastic rod and the specimen during stress wave propagation, and disregarding other energy dissipation mechanisms, it is assumed that the kinetic energy of the impact rod is entirely converted into the energy of the incident wave. The energy absorbed and dissipated by the specimen during impact-induced failure is then:


$$W_{S}=W_{I}-W_{R}-W_{T}$$ (4)

**Fig 31:**
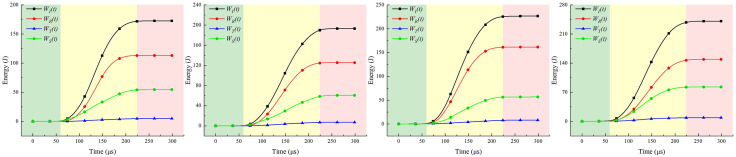
Energy-time curve of coal-rock composite under 0.3 MPa gas pressure. **(a)** C:R = 1:0. **(b)** C:R = 2:1. **(c)** C:R = 1:1. **(d)** C:R = 1:2.

**Fig 32:**
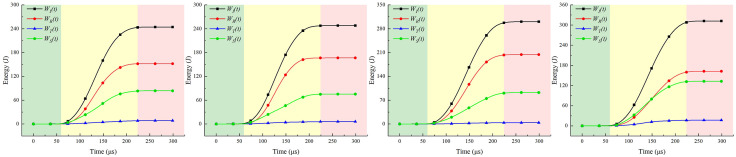
Energy-time curve of coal-rock composite under 0.5 MPa gas pressure. **(a)** C:R = 1:0. **(b)** C:R = 2:1. **(c)** C:R = 1:1. **(d)** C:R = 1:2.

**Fig 33:**
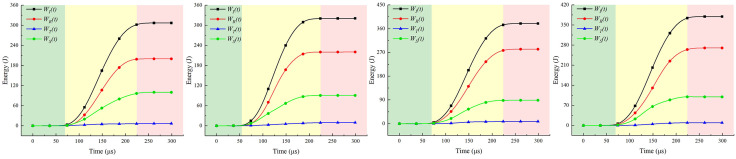
Energy-time curve of coal-rock composite under 0.7 MPa gas pressure. **(a)** C:R = 1:0. **(b)** C:R = 2:1. **(c)** C:R = 1:1. **(d)** C:R = 1:2.

Select the most favorable impact results from the three specimens for energy dissipation analysis. Figs 31–33 present the energy-time curves of the coal-rock composite under different impact pressures in the SHPB. It can be observed that all energy values increase with time, with each energy reaching its maximum nearly simultaneously in the final stage before stabilizing.

The curve pattern of energy dissipation is independent of the respective proportions within the coal-rock composite, though the amplitude of fluctuations varies. During the initial phase (0–50 μs), the energy trend and fluctuation levels are essentially identical. After 50 μs, the slopes of incident and reflected energy increase, while transmitted energy exhibits the smallest fluctuations, followed by dissipated energy. Based on the energy trend, the specimen’s dynamic compression energy-time curve can be broadly divided into three stages:

(1) Compression Energy Absorption Stage (0–50 μs): Upon stress wave arrival, the specimen enters a compressed state where energy is predominantly elastic with minimal dissipation.
(2) Energy Absorption and Dissipation Stage (50–225 μs): Stress wave energy exceeds the dynamic ultimate compressive strength of the coal-rock composite. Primary fractures develop and propagate, causing sustained energy absorption while generating some dissipation energy.
(3) Energy dissipation stage (225–300 μs): After reaching its dynamic ultimate compressive strength, the coal-rock composite fractures into irregular fragments, releasing internal energy.

Statistical analysis of stress-strain behavior and energy dissipation levels for coal-rock composites at different strengths is presented in Table 6.

**Table 6:** Energy Distribution statistics for coal-rock composites with different impact strengths.

Coal-rock ratio	Impact strength /MPa	Peak stress /MPa	Max. strain /*ε*	Incident energy *W*_*I*_/J	Reflected energy *W*_*R*_/J	Transmitted energy *W*_*T*_/J	Absorbed energy *W*_*S*_/J	Percentage of dissipated energy *R*
C:R = 1:0	0.3	7.5	0.00437	77.80	48.42	6.08	26.31	0.338
C:R = 2:1		9.6	0.0049	95.63	68.78	3.02	23.83	0.249
C:R = 1:1		11.0	0.00539	194.17	102.51	6.12	85.53	0.44
C:R = 1:2		13.9	0.00618	216.34	129.17	13.46	73.71	0.341
C:R = 1:0	0.5	8.9	0.00518	126.49	89.50	4.06	32.94	0.26
C:R = 2:1		11.5	0.00577	162.17	100.67	8.35	53.15	0.328
C:R = 1:1		13.3	0.00648	214.48	133.39	7.94	73.15	0.341
C:R = 1:2		15.9	0.00723	307.17	182.85	18.11	106.21	0.346
C:R = 1:0	0.7	12.4	0.00554	265.95	186.08	13.55	66.32	0.249
C:R = 2:1		14.0	0.00626	208.71	112.84	8.71	87.15	0.418
C:R = 1:1		16.0	0.0074	259.57	148.07	17.88	93.61	0.361
C:R = 1:2		19.3	0.00772	326.87	212.87	18.18	95.82	0.293

As shown in Table 6, the incident energy, reflected energy, and transmitted energy of the specimens gradually increase with rising impact pressure. Under identical impact pressure conditions, due to the significantly higher mechanical parameters (such as density and hardness) of rock compared to coal samples, coupled with the softening effect of water, parameters measured increase with higher rock content. At low impact intensity (0.3 MPa), the incident energy across different coal-to-rock ratios ranged from 172.64 to 240.89 J, with the dissipated energy proportion fluctuating between 0.249 and 0.44. When the impact pressure increased to 0.7 MPa, raising the rock proportion from C:R = 1:0 to C:R = 1:2 caused the incident energy to rise from 265.95 J to 326.87 J—an increase of approximately 60.92 J—with the dissipation energy ratio ranging from 0.249 to 0.418. The degree of dissipation in the sample is primarily influenced by its inherent physical and chemical properties. The magnitude of the impact pressure had no significant effect on the degree of dissipation.

## 5. Control measures for mitigating coal-rock dynamic disasters

### 5.1. Pre-splitting blasting with coordinated roof control technology

**Fig 34:**
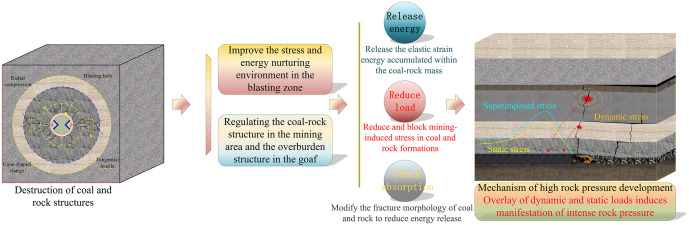
Model of energy release and structural regulation mechanism of deep hole blasting in top plate.

Based on the mechanism of strong rock pressure disasters in directly overlying hard roof strata, a mechanism model for vibration energy release and structural control against rock bursts during longwall mining was proposed. This model employs deep-hole pre-splitting blasting to regulate static and dynamic loads, as illustrated in Fig 34. Based on the principle of dynamic and static load superposition in strong mining pressure events, deep-hole pre-splitting blasting disrupts the coal-rock structure around blast holes, releasing accumulated internal energy. This improves the stress environment and energy accumulation conditions within the blasting zone. Within the pre-splitting zone formed during coal seam extraction, load reduction and vibration mitigation are achieved by regulating the structural integrity of the working roof system and the overburden structure in the goaf, thereby preventing strong mining pressure.

**Fig 35:**
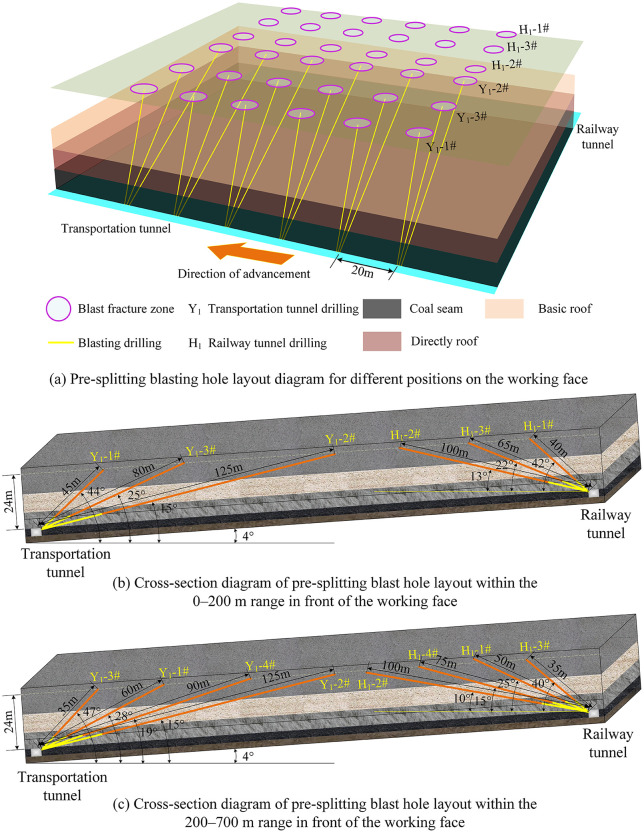
Schematic layout of pre-cracking blasting drill holes at different locations in the working face. **(a)** Pre-splitting blasting hole layout diagram for different positions on the working face. **(b)** Cross-section diagram of pre-splitting blast hole layout within the 0–200 m range in front of the working face. **(c)** Cross-section diagram of pre-splitting blast hole layout within the 200–700 m range in front of the working face.

To mitigate the impact of deep-hole blasting on the coal face, pre-splitting of the overlying hard rock strata is required in advance. This facilitates the timely collapse of the hard rock strata into the goaf after face advancement, thereby reducing the breakage strata distance of the hard rock. Such roof blasting serves as a means to control the manifestation of strong mining pressure by regulating the overburden structure. Considering the varying roof conditions during the development of the two crosscuts in the 11129 working face, deep-hole pre-splitting blasting was implemented in sections for the directly overlying hard roof. Given the coal seam dip angle variation between 2° and 6°, blast holes were designed based on an average dip angle of 4° to accommodate this variation. As the working face advances continuously and the coal seam dip angle changes, the blast hole angles can be appropriately adjusted (with a lateral deviation of 1° to 2°). The pre-splitting blasting plan for different locations in the 11129 working face is shown in Fig 35(a), with all parameters listed in Tables 7 and 8.

**Table 7:** Parameters of pre-cracking and blasting drill holes in the range of 0-200 m in front of the work.

Hole number	Hole length /m	Elevation angle /°	Hole size /mm	Charge length /m	Charge quantity /kg	Seal length /m	Detonator Section	Gun head Number
Y1-1	45	44	94	35	115.5	10	1	1
Y1-2	125	15	94	85	280.5	40	2	1
Y1-3	80	25	94	60	198	20	3	1
H1-1	40	42	94	30	99	10	1	1
H1-2	100	13	94	70	231	30	2	1
H1-3	65	22	94	45	148.5	20	3	1

**Table 8:** Parameters of pre-cracking and blasting drill holes in the range of 200-700 m in front of the work.

Hole number	Hole length /m	Elevation angle /°	Hole size /mm	Charge length m	Charge quantity /kg	Seal length /m	Detonator Section	Gun head Number
Y2-1	60	28	94	45	148.5	15	1	1
Y2-2	125	15	94	95	313.5	30	1	1
Y2-3	35	47	94	25	82.5	10	2	1
Y2-4	90	19	94	70	231	20	2	1
H2-1	50	25	94	35	115.5	15	1	1
H2-2	100	10	94	70	231	30	1	1
H2-3	35	40	94	25	82.5	10	2	1
H2-4	75	15	94	55	181.5	20	2	1

Pre-splitting blast holes are arranged in both roadways within the 0–200 m range ahead of the coal face, as shown in Fig 35(b). The first set of blast holes is positioned 12 m from the coal face. Starting from the second set, the inter-set spacing between each subsequent set and the preceding one is 20 m. Each set comprises three independently drilled holes at different angles, with a horizontal spacing of 1.5 m between each hole and alternating long and short holes. After loading and plugging every two sets of boreholes in the haulage or track drift, simultaneous detonation is possible. Parameters such as charge length and plugging length for the blast holes are detailed in Table 7.

Pre-splitting blast holes are arranged within a 200–700 m range ahead of the coal face, as shown in Fig 35(c). The group spacing for pre-splitting blast holes in both longwall roadways is increased to 30 m. Each group consists of four independently drilled holes at different angles, with a horizontal spacing of 1.5 m between holes within a group and alternating long and short holes. Parameters such as charge length and plug length for the blast holes are detailed in Table 8.

### 5.2. Study on the effect of pre-splitting blasting in coordinated roof control

**Fig 36:**
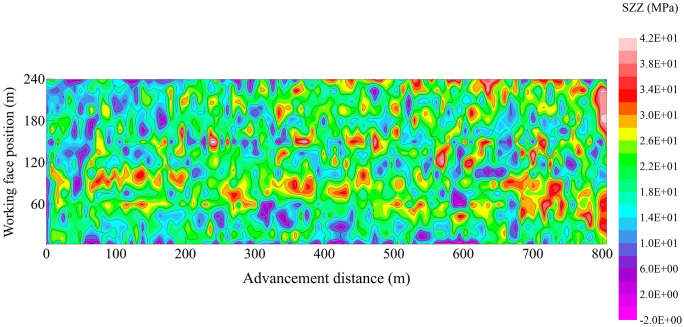
Cloud map of mine pressure stress distribution at the site of working face.

To further illustrate the overall stress distribution pattern of the mining area in a more intuitive and numerically quantifiable manner, pressure data from the hydraulic supports inclined toward the mining face were extracted. By summarizing and analyzing the stress conditions at different locations during face mining operations, a mining pressure stress map of the mining area was created, as shown in Fig 36.

The distribution of rock pressure in the working face at different distances from the mining area is shown in Fig 36. The contour map indicates that prior to the initial breakage (where the initial breakage step length plus the cutter length totaled 50 m), the overall pressure values in the working face were relatively low. The pressure between 80 and 120 m was higher compared to other locations, with a peak stress of 31.2 MPa. During the initial roof failure, stress values near the lower face (0–80 m) were elevated, ranging from 18.8 to 22.7 MPa overall. Supports in the middle section of the working face experienced lower stresses, while individual supports at the upper face recorded pressures of 18.9 MPa. Overall, pressure values in the upper section were lower than those in the lower supports. Following the initial cyclic failure of the roof, the pressure on the supports in the middle and lower sections of the working face showed an upward trend, with the peak support pressure reaching 29.3 MPa. The pressure curve of the working face supports exhibited a pattern of higher values in the middle and lower sections and lower values at both ends. The minimum support stress recorded was 13.1 MPa, occurring in the middle and lower sections of the working face. The pre-splitting blast range for the working face roof extends from 0 to 700 m. During continuous advancement, the support pressure within the 50–110 m range of the working face is relatively higher than in other locations, with a longer duration of pressure application. As the working face advanced to 670 m, the rear goaf area gradually expanded. Overlying medium-to-high-level caprock layers successively experienced rotational subsidence, exerting pressure onto the goaf and increasing the load-bearing stress on the solid coal-rock strata in front of the coal face. This manifested as a sustained increase in hydraulic support pressure within the mining area. At this stage, support pressure values near both ends of the working face exceeded those in the central section, with overall values ranging from 23.6 to 31.5 MPa. When the working face advances beyond the pre-splitting blast zone (greater than 700 m). At the 730 m advance point, the overall pressure values of the support increased significantly, with the central support pressure rising from 14.7–18.3 MPa to 30.0–32.3 MPa. Simultaneously, the pressure values near the upper and lower end supports also increased correspondingly, with the maximum hydraulic support pressure reaching 35.3 MPa. It is evident that roof pre-splitting markedly reduces overall support pressure, thereby improving the working environment for hydraulic supports in the working face. This ensures safe and efficient coal seam recovery.

## 6. Conclusions

(1) The hard sandstone roof exhibits load-bearing characteristics of large span and minimal deformation during mining operations, providing temporary support for the overlying soft rock strata. Field monitoring revealed an initial roof collapse step size of approximately 45 meters, followed by periodic collapses with step sizes ranging from 10 to 30 meters. The collapse morphology ultimately evolved into a typical trapezoidal structure. This provides insights into the overall failure patterns of deep composite rock mass systems.
(2) During coal seam mining, energy undergoes accumulation and release. When the roof has not reached its critical span, the hard rock strata primarily bear and accumulate energy. Upon reaching its limit, the rock strata fracture, transferring the load and releasing energy into the goaf. This reveals the intrinsic mechanism of energy-driven evolution in the fracture morphology of composite structures.
(3) Dynamic experiments demonstrate that the fracture evolution of coal rock specimens follows a progressive damage pattern of “initiation-development-fracture.” Its energy response can be divided into three stages: compression energy absorption, energy dissipation absorption, and energy dissipation. The higher the lithofacies proportion (high density, high hardness), the more outstanding its overall strength and energy indicators perform, confirming the influence of material heterogeneity on macroscopic mechanical behavior.
(4) Based on the established peak stress damage patterns of coal and rock under combined static and dynamic loading, this study proposed and implemented a cluster fan-shaped borehole pre-splitting blasting scheme as an active method for structural weakening and performance regulation. This technique effectively shortened the natural breakage stride length in hard rock strata. Both numerical simulations and field rock pressure observations confirmed that this method successfully controlled the timing and structure of roof failure, achieving the anticipated pressure relief and control effects. For large-scale coal bodies at the site, the research findings can to a certain extent reflect the general failure patterns of coal bodies, providing an experimental foundation for in-depth studies on crack propagation calculations in coal bodies.
